# Inferring phylogenies with incomplete data sets: a 5-gene, 567-taxon analysis of angiosperms

**DOI:** 10.1186/1471-2148-9-61

**Published:** 2009-03-17

**Authors:** J Gordon Burleigh, Khidir W Hilu, Douglas E Soltis

**Affiliations:** 1 National Evolutionary Synthesis Center (NESCent), Durham, NC 27705, USA; 2 Department of Botany and Zoology, University of Florida, Gainesville, FL 32611, USA; 3 Department of Biological Sciences, Virginia Tech, Blacksburg, VA 24061, USA

## Abstract

**Background:**

Phylogenetic analyses of angiosperm relationships have used only a small percentage of available sequence data, but phylogenetic data matrices often can be augmented with existing data, especially if one allows missing characters. We explore the effects on phylogenetic analyses of adding 378 *matK *sequences and 240 26S rDNA sequences to the complete 3-gene, 567-taxon angiosperm phylogenetic matrix of Soltis et al.

**Results:**

We performed maximum likelihood bootstrap analyses of the complete, 3-gene 567-taxon data matrix and the incomplete, 5-gene 567-taxon data matrix. Although the 5-gene matrix has more missing data (27.5%) than the 3-gene data matrix (2.9%), the 5-gene analysis resulted in higher levels of bootstrap support. Within the 567-taxon tree, the increase in support is most evident for relationships among the 170 taxa for which both *matK *and 26S rDNA sequences were added, and there is little gain in support for relationships among the 119 taxa having neither *matK *nor 26S rDNA sequences. The 5-gene analysis also places the enigmatic *Hydrostachys *in Lamiales (BS = 97%) rather than in Cornales (BS = 100% in 3-gene analysis). The placement of *Hydrostachys *in Lamiales is unprecedented in molecular analyses, but it is consistent with embryological and morphological data.

**Conclusion:**

Adding available, and often incomplete, sets of sequences to existing data sets can be a fast and inexpensive way to increase support for phylogenetic relationships and produce novel and credible new phylogenetic hypotheses.

## Background

Molecular data have had an enormous impact on angiosperm phylogenetic hypotheses (e.g. [1-5]), and the abundance of new sequence data provides the potential for further resolving angiosperm relationships. Still, molecular phylogenetic studies across all angiosperms have utilized only a small fraction of the available sequence data. While GenBank currently contains over 1.7 million core nucleotide sequences from angiosperms, with over 160,000 of these being from often phylogenetically useful plastid loci [6], few phylogenetic analyses of angiosperms have included more than a thousand sequences. We examine whether augmenting existing plant data matrices with incomplete data assembled from publicly available sources can enhance the understanding of the backbone phylogenetic relationships across angiosperms.

The sampling strategies of phylogenetic studies across angiosperms demonstrate a tradeoff between taxonomic sampling and the number of gene sequences per taxon. On one extreme, phylogenetic analyses using single genes such as *rbcL *[7,8], 18S rDNA [9], and *matK *[10] have sampled hundreds, or even thousands [8], of taxa. In some cases, analyses of single genes have provided strong support for many angiosperm relationships (e.g. [10]), but other parts of the single-gene trees have been unresolved, in disagreement with other single gene trees (albeit often without strong support), or simply anomalous (e.g. [7]). On the other extreme, the advent of chloroplast genome sequencing has led to phylogenetic analyses of angiosperms using sequences from up to 81 genes, although often with limited taxon sampling [11-17]. The limited taxon sampling can have undesirable effects on phylogenetic inferences from complete chloroplast genome sequences, as well as other large molecular data sets (see [13,14,18,19]). Other studies have used sampling strategies that attempt to compromise between taxon and gene sampling. For example, Qiu et al. [20,21] used 5 genes and 105 taxa, and later studies have used 9 genes and 100 taxa [22] or 8 genes and 162 taxa [23]. These studies generally have focused on the basal angiosperm splits, and more comprehensive taxon sampling is necessary to address backbone relationships throughout the angiosperms. The most comprehensive taxon sampling across angiosperms using multiple genes includes a study using *atpB *and *rbcL *[24] and a 567-taxon study with 18S rDNA, *atpB*, and *rbcL *[1-3].

While more data are needed to further resolve backbone relationships in angiosperms, it is not clear what the most efficient sampling strategies for adding new data would be. Previous analyses of molecular data across all angiosperms mostly used complete or nearly complete data matrices (but see [25,26]), in which all taxa have sequences from all, or nearly all, genes. Often it is possible to increase the number of taxa or genes in a phylogenetic matrix greatly using existing data by allowing small amounts of missing data (e.g. [27]). Furthermore, recent simulation studies have demonstrated that in some cases the total amount of data in a phylogenetic matrix may determine the performance of a phylogenetic analysis more than the percentage of missing data [28]. This suggests that adding sequences from genes with incomplete taxonomic coverage to existing complete data matrices can, at least in some cases, improve the phylogenetic inference. Generating large, complete data matrices can be both logistically daunting (e.g., assembling DNA samples from all taxa) and prohibitively expensive. In contrast, there is a wealth of publicly available sequence data that can be readily assembled into phylogenetic data matrices, providing a relatively fast and inexpensive way to augment existing data sets and possibly improve our phylogenetic inferences.

We explore the effect of adding existing plastid *matK *and nuclear 26S rDNA sequences to the 3-gene (18S rDNA, *atpB*, and *rbcL*) 567-taxon matrix of P. Soltis et al. ([1], see also [2,3]). Both *matK *and the nuclear 26S rDNA have been informative in large-scale angiosperm phylogenetic studies (e. g., *matK*: [10,22,23]; 26S rDNA: [29-32]), and consequently, sequence data from both genes are available for many angiosperm taxa. We assembled the available 26S rDNA and *matK *sequences from taxa represented in the 3-gene matrix and compared results from maximum likelihood phylogenetic analyses of the 3-gene 567-taxon complete matrix with the 5-gene 567-taxon incomplete matrix. Specifically, we examine if and how augmenting complete data sets with incomplete data affects our view of angiosperm phylogeny.

## Results

### Data set

**Figure 1:**
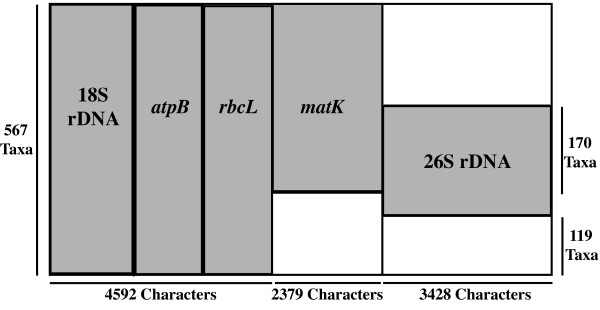
**Diagram representing the distribution of data in the total 5-gene data matrix**. All taxa in the matrix contain sequences from the first 3 genes (18S rDNA, *atpB*, and *rbcL*), 378 taxa have *matK *sequences, and 240 taxa have 26S rDNA sequences. Only 170 taxa have sequences from both *matK *and 26S rDNA, and 119 taxa have no *matK *or 26S rDNA sequences.

We performed analyses on a 3-gene and a 5-gene data matrix that have the same set of 567-taxa (Fig. 1). In the 3-gene (18S rDNA, *atpB*, and *rbcL*), matrix, all taxa have sequences from all genes. The matrix is 4592 characters long and it contains 2.9% missing data, representing indels or small sections of missing gene sequences. The 5-gene matrix comprises the available *matK *and 26S rDNA sequences concatenated to the 3-gene matrix. The accession numbers for the matK and 26S rDNA sequences, as well as the 5-gene character matrix, are included as additional files (see Additional files 1, 2 and 3). In the 5-gene matrix, 170 (29.9%) taxa have sequences from all 5 genes, and 119 (20.9%) taxa have data from only the original 3-genes (18S rDNA, *atpB*, and *rbcL*; Fig. 1). In total 47.0% of the *matK *and 26S rDNA alignments are missing data, largely due to missing whole gene sequences, and overall, 27.5% of the cells in the 5-gene 567-taxon matrix are missing data.

### Phylogenetic Analyses

**Figure 2:**
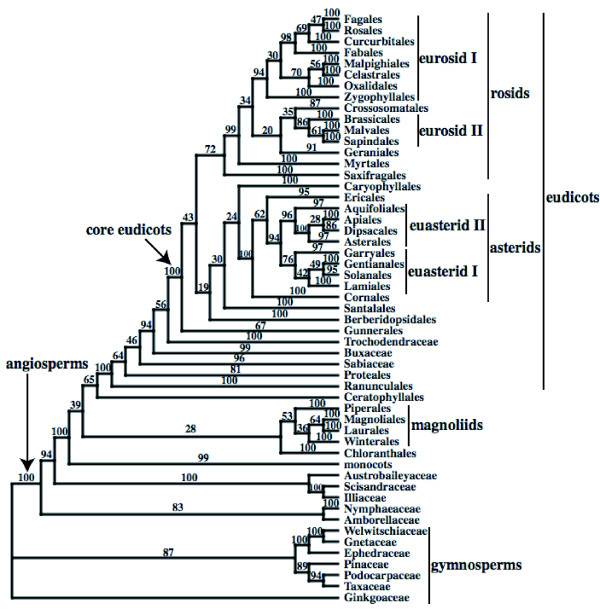
**Summary of the majority rule consensus from the 3-gene (18S rDNA, *atpB*, and *rbcL*) ML analysis**. Names of the orders and informal names follow APG II [4] and Soltis et al. [2,3], with *Hydrostachys *in Cornales. Numbers above the branches are bootstrap percentages. This tree was rooted using all gymnosperm taxa as outgroups.

**Figure 3:**
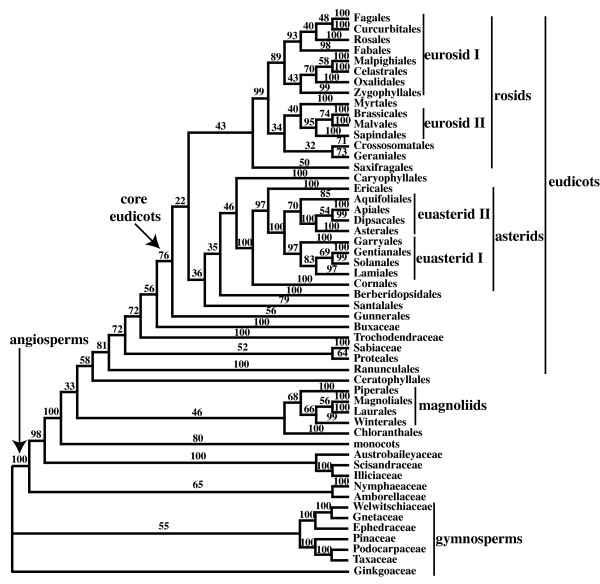
**Summary of the majority rule consensus from the 5-gene (18S rDNA, *atpB*, *rbcL, matK*, and 26S rDNA) ML analysis**. Names of the orders and informal names follow APG II [4] and Soltis et al. [2,3], with *Hydrostachys *in Lamiales. Numbers above the branches are bootstrap percentages. This tree was rooted using all gymnosperm taxa as outgroups.

The bootstrap trees from the 5-gene and 3-gene analyses are included as additional data (see Additional files 4 and 5). The 5-gene ML bootstrap analysis produced higher levels of support than the 3-gene ML bootstrap analysis. First, the analysis of the 5-gene matrix produced more clades with high bootstrap support score (Table 1, Figs. 2 and 3; Additional files 6 and 7). Also, the average quartet similarity among bootstrap trees was higher in the 5-gene analysis than the 3-gene analysis (0.963 vs. 0.934; Table 1). Still, bootstrap support for some clades, decreased. For example, bootstrap support for monocot, eudicot, and core eudicot clades decreased from 99% or 100% in the 3-gene analysis to between 76% and 81% the 5-gene analyses (Figs. 2 and 3; Additional files 6 and 7). There are few major differences in the topologies of the 3-gene and 5-gene ML bootstrap 50% majority rule consensus trees, and these topologies are largely consistent with results from previous 3-gene parsimony [1,2] and Bayesian analyses [3]. Therefore, we include the full bootstrap consensus trees as additional files (see Additional files 6 and 7).

**Table 1:** Summary of support for 3-gene and 5-gene 567-taxon maximum likelihood bootstrap analyses.

	**Clades with Bootstrap Support**					
	**100%**	**≥95%**	**≥90%**	**≥70%**	**≥50%**	**Ave. Quartet Similarity**
	
5-gene ML	223	290	318	409	487	0.963
3-gene ML	190	273	298	378	461	0.934

The first 5 columns show the number of clades (out of a possible 564) that have the specified level of bootstrap support. The last column shows the average percent of quartets (4-taxon unrooted trees) that are identical among bootstrap trees.

**Figure 4:**
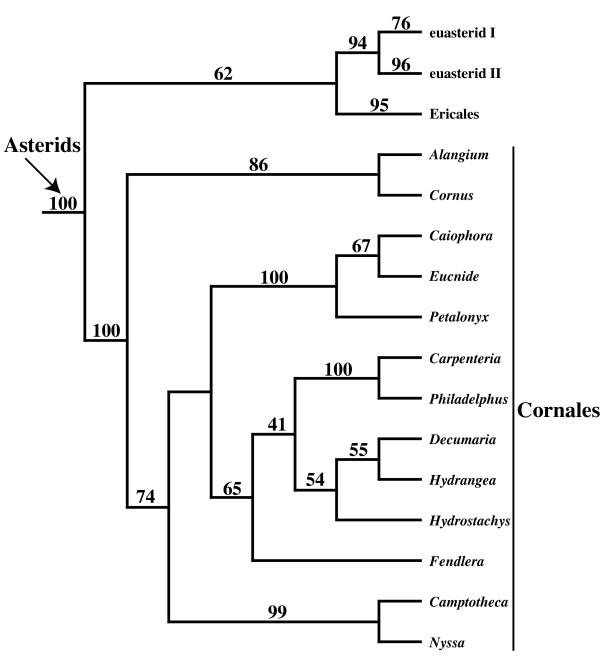
**Detail of the position of *Hydrostachys *within Cornales in the majority rule consensus from the 3-gene ML analysis**.

**Figure 5:**
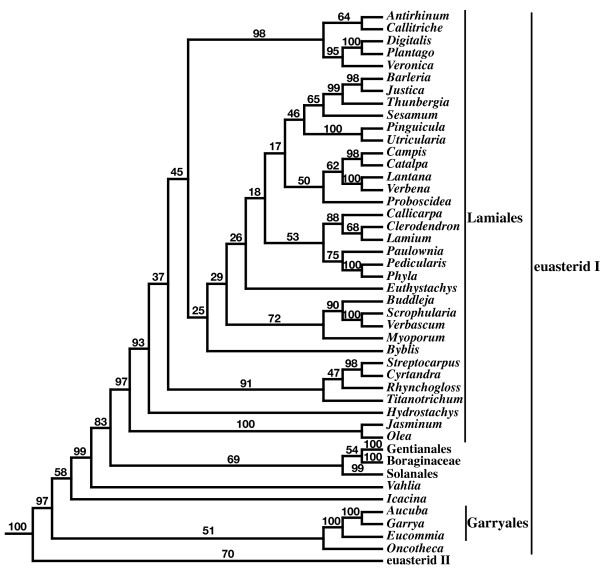
**Detail of the position of *Hydrostachys *within Lamiales in the majority rule consensus from the 5-gene ML analysis**.

Perhaps the biggest single change in topology between the 5-gene and 3-gene ML analyses was the position of *Hydrostachys *(Figs. 4 and 5), which has a *matK *but no 26S rDNA sequence in the 5-gene matrix. In the 3-gene ML analysis, there was 100% bootstrap support for placing *Hydrostachys *within Cornales (Fig. 4), which is sister to all remaining asterids (the Ericales + euasterid I and II clade) (Figs. 2 and 4). In contrast, the 5-gene ML analysis placed *Hydrostachys *in Lamiales (Fig. 5), within the euasterid I clade (Figs. 3 and 5). Although there was 97% bootstrap support for the Lamilaes clade containing *Hydrostachys *in the 5-gene analysis, the placement of *Hydrostachys *within Lamiales was largely unresolved (Fig. 5). Within Lamilaes, there was 93% support for a clade of all taxa (including *Hydrostachys*) except *Olea *and *Jasminum *(Fig. 5).

The average taxon quartet similarity, the average quartet similarity between all pairs of bootstrap trees for only the quartets that contain the taxon, was higher for every taxon in the 5-gene analysis than the 3-gene analysis (not shown). In the 5-gene bootstrap analyses, the taxa with the lowest average taxon quartet similarity values, or whose position were least supported (or vary most) among bootstrap trees, are largely from early diverging eudicot clades (e.g., Santalales, Dilleniaceae, and Berberidopsidales), and the core eudicots (Gunnerales) (Table 2).

**Table 2:** Taxa with the least support in the 5-gene ML analysis.

	**Taxa**	**Ave. Taxon Quartet Similarity**
1	*Dendrophthora*	0.779
2	*Cercidiphyllum*	0.824
3	*Tetracera*	0.827
4	*Dillenia*	0.828
5	*Myrothamnus*	0.863
6	*Berberidopsis*	0.869
7	*Aextoxicon*	0.875
8	*Schoepfia*	0.889
9	*Eubrachion*	0.889
10	*Santalum*	0.889

The average taxon quartet similarity is the average percentage of all quartets (4-taxon unrooted trees) that include the taxon that are identical among bootstrap trees.

### Effects of adding loci with incomplete taxonomic coverage

We further examined the effects of adding incomplete data sets by comparing the support within the 3-gene and 5-gene, 567-taxon analyses for relationships among the 170 taxa having all five gene sequences (the original three genes plus both 26S rDNA and *matK *sequences) and the 119 taxa having neither 26S rDNA nor *matK *sequences (Fig. 1). To do this, we examined reduced consensus bootstrap trees (e.g. [33]) that included just the 170 taxa with data from all 5 genes and also that included only the 119 taxa with data from only 3 genes (missing both *matK *and 26S rDNA; Figure 1). The reduced consensus trees were made by pruning all the taxa except the specified sets of 170 or 119 from the 567-taxon bootstrap trees and then making a majority rule consensus from the pruned bootstrap trees. Adding both *matK *and 26S rDNA resulted in greater increases in support among relationships between the 170 taxa with 5 genes than among the 119 taxa with 3 genes (Table 3). There were increases in the number of highly supported clades in the reduced consensus tree containing the 170 taxa with 5 genes, and the average quartet similarity among the 170-taxon bootstrap trees increases by 0.044 with the addition of *matK *and 26S rDNA (Table 3). In contrast, the number of clades with 100%, ≥95%, and ≥90% bootstrap support in the 119-taxon reduced consensus is lower in the 5-gene than in the 3-gene analysis, and the average quartet similarity among the 119-taxon bootstrap trees increases by only 0.010 with the addition of *matK *and 26S rDNA (Table 3).

**Table 3:** Summary of the support for relationships among the 170 taxa with sequences from 5 genes and the 119 taxa with sequences from 3 genes.

	**Clades With Bootstrap Support**					
	**100%**	**≥95%**	**≥90%**	**≥70%**	**≥50%**	**Ave. Quartet Similarity**
	
5-Gene 170-taxa	89	103	109	128	146	0.946
3-Gene 170-taxa	70	93	99	119	137	0.902
Change	19***	10	10	9	9	0.044***
						
5-Gene 119-taxa	39	51	56	82	94	0.952
3-Gene 119-taxa	49	54	61	77	87	0.942

Change	-10***	-3*	-5**	5	7	0.010***

The support values were obtained from reduced consensus trees, which were made by pruning all but either the selected 170 or 119 taxa from the bootstrap trees and making a consensus from the pruned trees. The first 5 columns show the number of clades in the reduced consensus trees that have the specified level of bootstrap support. The last column shows the average percent of quartets (4-taxon unrooted trees) that are identical between the pruned bootstrap trees. We tested if the observed changes in the bootstrap support or average quartet similarity are greater in the 170 taxa with 5-genes than they would be from a random sample of 170 taxa. Similarly, we tested if the changes in support are less in the 119 taxa with 3 genes than they would be from a random sample of 119 taxa. Significant results are noted with "*" symbols (* p ≤ 0.05, ** p ≤ 0.01, *** p ≤ 0.005).

We next tested the hypothesis that the observed changes in bootstrap and average quartet similarity scores for relationships among the 170 taxa with 5 genes were *greater *than we would expect from a random sample of 170 taxa. We did this by comparing the observed changes in bootstrap and quartet similarity scores from the 170 taxa with 5 genes to changes in support for relationships among 200 sets of 170 randomly selected taxa. We found significantly greater than expected increases in the number of clades with 100% bootstrap support and in the average quartet similarity (p ≤ 0.005; Table 3). Similarly, we tested to see if the changes in support for relationships among the 119 taxa with 3 genes were *less *than we would expect from a random sample of 119 taxa. We examined changes in support from 200 sets of 119 randomly selected taxa and found significantly greater than expected decreases in the number of clades with 100%, ≥95%, and ≥90% bootstrap support (p ≤ 0.005, 0.05, and 0.01 respectively; Table 3) and in the average quartet similarity (p ≤ 0.005; Table 3).

Finally, we compared changes in the average taxon quartet similarity among bootstrap trees from the 3-gene to the 5-gene analysis for all taxa, just the 170 taxa with data from 5 genes, and just the 119 taxa with data from 3 genes. The mean change in the average taxon quartet similarity for the 170 5-gene taxa was greater than the mean across all taxa, and the mean change for the 119 3-gene taxa was less than that across all taxa (Table 4). The mean change in the average taxon similarity for the 170 5-gene taxa is *greater *than we would expect from a random sample of 170 taxa (p ≤ 0.001; Table 4), and the mean change for the 119 3-gene taxa is *less *than we would expect from a random sample of 119 taxa (p ≤ 0.001; Table 4).

**Table 4:** Summary of average taxon quartet similarity scores across bootstrap trees.

	**Mean Ave. Taxon Quartet Similarity**		
	**3-Gene ML**	**5-Gene ML**	**Ave. Change**
	
All taxa	0.937	0.958	0.021
170-taxa	0.928	0.954	0.026***
119-taxa	0.941	0.957	0.016***

The average taxon quartet similarity is the average proportion of quartets (4-taxon unrooted trees) that include a particular taxon that have the same topology among bootstrap trees. The "170 taxa" are the taxa with sequences from all 5 genes, while the "119 taxa" are those with sequences from only the original three genes (18S rDNA, *atpB*, *rbcL*). "Ave. Change" shows the difference in the mean of the average taxon quartet similarity between the 3-gene and 5-gene ML bootstrap analyses. We tested if the average change among the 170 taxa is significantly *greater *than would be expected by randomly selecting without replacement 1000 sets of 170 taxa. Similarly, we tested if the average change among the 119 taxa with 3 genes is significantly *less *than would be expected by randomly selecting without replacement 1000 sets of 119 taxa. Both tests were highly significant (*** = p ≤ 0.001).

## Discussion

Augmenting existing data sets with other available sequence data can enhance our understanding of angiosperm phylogeny, even if the new sequence data are incomplete. Although adding available *matK *and 26S rDNA sequences to the complete 3-gene angiosperm data set increases the overall percentage of missing data in the character matrix from 2.9% to 27.5%, analyses with the new sequences provide higher overall levels of support than analyses of the original 3 genes alone (Figs. 2 and 3, Table 1). The gains in support are especially evident in the relationships among the 170 taxa with both *matK *and 26S rDNA sequences (Tables 3 and 4). In contrast, there is little increase in support for relationships among the 119 taxa that do not have *matK *or 26S rDNA sequences (Tables 3 and 4). This relationship between the amount of data and support suggests that, although incorporating available sequence data can increase support for much of the tree, a complete understanding of the relationships among all taxa likely will require more complete sampling. Specifically, among angiosperms, the average taxon quartet scores indicate that more data is especially needed to resolve the early diverging branches within eudicots (Table 2).

The gains in bootstrap and quartet similarity support resulting from the new *matK *and 26S rDNA sequences mostly are incremental, and they do not notably alter our view of most angiosperm relationships. One major exception is the placement of *Hydrostachys*, an aquatic genus from Madagascar and southern Africa, which is in Cornales in the 3-gene ML analyses and the Lamiales in the 5-gene ML analyses (Fig. 4). The placement of *Hydrostachys *within Lamiales also is strongly supported by maximum parsimony bootstrap analyses of the 5-gene matrix (Burleigh, unpublished). This new result appears to be driven by the *matK *sequences, and an ML analysis of just our *matK *data also places *Hydrostachys *in Lamiales (not shown). The new *Hydrostachys matK *sequence was originally included as an outgroup for an analysis of Loasaceae [34], and it is from not only the same species but the same vouchered specimen that was used to obtain the data for the other three genes [Schatz et al. 3413 (MO)].

While the 5-gene analysis provides a new perspective on the placement of Hydrostachys, its placement has always been enigmatic. Xiang et al. [35] and Fan and Xiang [36] placed *Hydrostachys *among the earlier diverging Cornales lineages, but these studies did not sample extensively outside Cornales. A 3-gene analysis by Albach et al. [37] also placed *Hydrostachys *within in Cornales [38], but the authors also noted that *Hydrostachys *had long molecular branches and few morphological characters to support its placement in Cornales [38]. In contrast, placement of *Hydrostachys *within Lamiales is consistent with embryological [37,39] and floral morphological [40] data. Consequently, it was classified within Lamiales by Dahlgren [41], Cronquist [42], and Takhtajan [43]. Still, while the placement of *Hydrostachys *within Lamiales is intriguing and credible, we urge caution in interpreting this result. Additional molecular data, as well as analyses to examine the causes for the various molecular results and sources of phylogenetic error, are necessary to confirm the position of *Hydrostachys*.

Adding *matK *and 26S rDNA sequences with incomplete taxonomic sampling appears to be beneficial for the inference of the angiosperm phylogeny, but there are still troubling aspects of the 5-gene analysis. Perhaps foremost is the reduction of bootstrap support for a few well-accepted clades such as monocots, eudicots, and core eudicots (Fig. 3). In simulation, adding characters with missing data can decrease the probability of resolving the true phyogeny [44,45], and this is consistent with a reduction in bootstrap support. Furthermore, adding characters with missing data also can have a similar effect as reducing taxon sampling; that is, they can effectively increase the length of branches from sampled taxa and the proportion of characters that support erroneous topologies [44]. Taxonomic sampling can drastically affect the results of phylogenetic analyses (e.g. [46-48]), and sufficient taxonomic sampling is an especially relevant concern in analyses of angiosperm relationships [13,14,17]. Still, there is no obvious evidence of strong taxon sampling-like error in the 5-gene analysis. Each of the added genes had sequences from at least 240 taxa, so no parts of the character matrix had especially poor taxon sampling. Furthermore, bootstrap support for monocots, eudicots, and core eudicots are still at least 78% (Fig. 3), and, besides the placement of *Hydrostachys*, there are few strongly supported, major differences in the 5-gene and 3-gene results.

Our results suggest other directions for future phylogenetic research. In our study, we increased sampling by taking a fixed set of taxa and adding genes that had many sequences from these taxa. We might also expand our sampling by adding new taxa that have sequences from most or all of the 5-genes (e.g. [25]). This may ameliorate any potential problems associated with inadequate taxon sampling. We also note that conventional nonparametric bootstrapping methods (e.g. [49]) do not explicitly account for missing data, and it may be profitable to explore bootstrapping methods explicitly designed for incomplete data sets (e.g. [50]).

## Conclusion

Although there has been much recent progress in elucidating angiosperm phylogeny, there are still many unresolved relationships that are critical to understanding the angiosperm evolution. New data are needed; yet assembling new, complete data sets across all angiosperms is both extremely time-consuming and expensive. While most major analyses of angiosperm relationships have used complete or nearly complete data sets, this study demonstrates that exploring new ways to exploit existing angiosperm data can be a fast, cost-effective, and informative complement to more conventional systematic efforts to sequence new genes.

## Methods

### Taxon sampling and data sets

The original 3-gene (nuclear 18S rDNA, plastid *atpB *and *rbcL*) matrix includes 567 taxa, and the alignment is 4592 characters in length. The set of excluded characters, and thus the total length of the alignment, differs slightly from previous analyses [[1-3]; see Additional file 3]. Each terminal "taxon" in the 567-taxon matrix represents a single genus, and in some cases, gene sequences from congeneric species were combined (see [2]). (The original matrix contains two species from the genus "*Saxifraga*", but *Saxifraga integrifolia *is now *Micranthes integrifolia*). Thus, we also added data from some congeneric species for *matK *and 26S rDNA (see Additional files 1 and 2). We first searched GenBank for *matK *sequences from genera that were included in the original 567-taxon matrix. If multiple *matK *sequences were found from a particular genus, we chose the longest one. Consequently, some of the sequences include sections of the *trnK *intron regions that flank *matK*. Additionally, we added a new *matK *sequence from *Gunnera*. In total, we had at least partial *matK *sequences from 378 of the 567 terminal taxa (Fig. 1; Additional file 2). The *matK *sequences were aligned using on the protocol of Hilu et al. [10], and the total *matK *alignment was 2379 characters in length (Fig. 1; Additional file 3). The 26S rDNA sequence data were taken from previously analyzed data sets [[30-32]; Additional file 1]. They were aligned using Clustal W [51] and further edited manually, deleting a few small sections in which we could not confidently determine the homology of characters across taxa. In total, 240 out of the original 567 taxa have 26S rDNA sequences, and the 26S rDNA alignment was 3428 characters in length (Fig. 1; see Additional file 3). We concatenated the *matK *and 26S rDNA sequences to the original 3-locus complete data matrix to generate the 5-locus data matrix (Fig. 1; Additional file 3). All phylogenetic analyses were performed on both the 3-gene (18S rDNA, *atpB*, and *rbcL*) 567-taxon data set, which has no missing gene sequences, and the 5-gene 567-taxon matrix, which includes taxa with missing gene sequences.

### Phylogenetic analyses

For both the 3-gene and 5-gene data matrices, we performed 100 maximum likelihood (ML) bootstrap replicates using GARLI v. 0.951 [52], which implements a genetic heuristic algorithm for the tree search. We relied on bootstrap support because 1) we are interested in directly assessing how adding characters with missing data affects sampling variance and 2) the 567-taxon data sets appear to have much trouble reaching stationarity in Bayesian analyses [3]. All tree searches started from a neighbor-joining topology [53] and otherwise used the default settings from GARLI. The likelihood function incorporated the general time reversible substitution model (GTR; [54]), which allows different substitution rates for each type of nucleotide substitution, with rate variation among sites estimated using a discrete gamma distribution with four rate categories [55] and a separate parameter for the percentage of invariable sites. The bootstrapped data sets were generated (sampled with replacement from the original data set) using HyPhy [56]. We found that the ML estimates of 567-taxon topology using GARLI occasionally differed, suggesting that that the GARLI tree search may get trapped in local optima. Therefore, for each bootstrap replicate, we performed 5 runs of GARLI and selected a tree with the highest likelihood across the five runs. We also performed maximum parsimony bootstrap analyses on the 3-gene and 5-gene data sets using a parsimony ratchet search strategy [57] implemented in PAUP* [58]. The overall bootstrap support was slightly lower than in the ML analyses, but the results were very similar. Therefore, we just focus on ML results for this paper.

### Comparison of tree topologies

We used two measures to compare levels of support from the 3-gene and 5-gene phylogenetic analyses. First, we simply compared the bootstrap support from the two analyses, specifically the number of partitions (clades in a rooted tree) that had bootstrap support of 100%, ≥95%, ≥90%, ≥70%, and ≥50%. However, the bootstrap support for each partition does not describe the support for relationships among all sets of taxa. Therefore, we also used a measure based on the quartet distance (e.g. [59]) to examine and compare the support in the 3-gene and 5-gene analyses in more detail.

A quartet is set of four taxa, and it represents the smallest unit of phylogenetic information in a tree. In a 567-taxon tree, there are over 4.26 billion possible quartets. The *quartet similarity *(or 1- quartet distance) is the percentage of all quartets with identical unrooted topologies in two trees. We first calculated the *average quartet similarity *between all pairs of bootstrap trees. We also quantified the phylogenetic support for each taxon in the bootstrap trees using quartet distances (e.g. [59]). To do this, we measured the *average taxon quartet similarity*, which, for a specified taxon, is the average quartet similarity between all pairs of bootstrap trees for only the quartets that contain the specified taxon. For example, in the 567-taxon tree, only just over 30 million of the 4.26 billion possible quartets involve any single taxon, like *Amborella*. The average taxon quartet similarity for *Amborella *measures the average quartet similarity among all pairs of bootstrap trees only for the ~30 million quartets that contain *Amborella*. Taxa whose positions vary among bootstrap trees will have lower average taxon quartet similarity scores than taxa with similar positions among bootstrap trees. For each set of bootstrap trees, there is a single average quartet similarity score, but there are 567 average taxon quartet similarity scores, one for each taxon. The quartet similarity scores were computed with QDist [60] and a series of perl scripts.

## Authors' contributions

This study was conceived and designed by all authors. DES provided initial 26S rDNA sequence alignments and a new *matK *sequence. JGB assembled the remaining *matK *sequences, which KWH aligned. JGB designed and performed the computational analyses and wrote the manuscript with help from KWH and DES. All authors read and approved the final manuscript.

## Supplementary Material

Additional file 1 **26S.accession.** Accession table for 26SrDNA sequences used in this study. Click here for file

Additional file 2 **matK.accession.** Accession table for *matK *sequences used in this study. Click here for file

Additional file 3 **5gene.** Text file in nexus format with the sequence alignment used for analyses in this study. Click here for file

Additional file 4 **3gene.AllMLBS.** Text file containing the bootstrap trees from the 3-gene maximum likelihood bootstrap analysis. Click here for file

Additional file 5 **5gene.AllMLBS.** Text file containing the bootstrap trees from the 5-gene maximum likelihood bootstrap analysis. Click here for file

Additional file 6 **3gene.MLBS.** PDF file with a figure showing the full majority rule consensus tree from the 3-gene maximum likelihood bootstrap analysis. Click here for file

Additional file 7 **5gene.MLBS.** PDF file with a figure showing the full majority rule consensus tree from the 5-gene maximum likelihood bootstrap analysis. Click here for file
