# Supplementary figures and images for: Inferring phylogenies with incomplete data sets: a 5-gene, 567-taxon analysis of angiosperms
Source: BMC Evol Biol. 2009 Mar 17;9:61. doi: 10.1186/1471-2148-9-61 (PMC2674047; doi:10.1186/1471-2148-9-61)

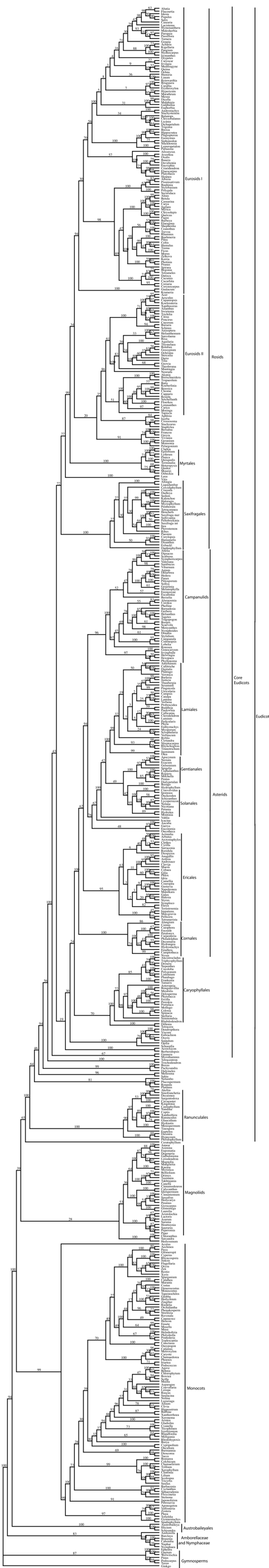

Supplement: Additional file 6 — 3gene.MLBS. PDF file with a figure showing the full majority rule consensus tree from the 3-gene maximum likelihood bootstrap analysis. [file 1471-2148-9-61-S6.pdf]

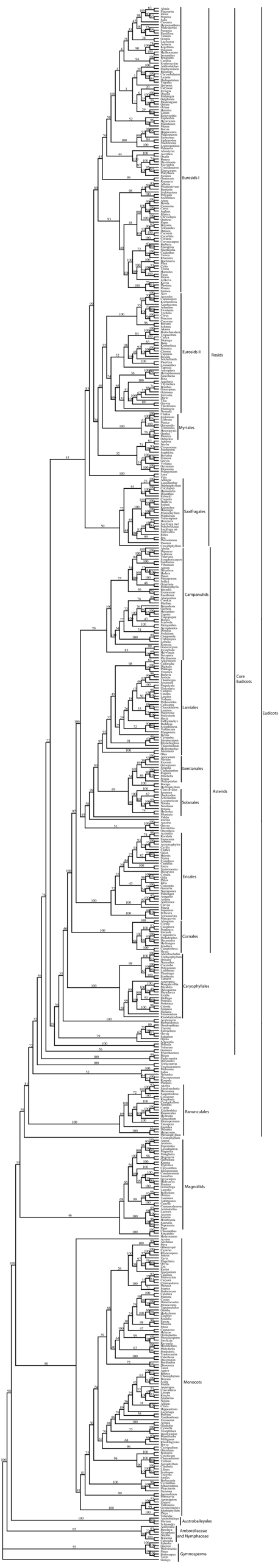

Supplement: Additional file 7 — 5gene.MLBS. PDF file with a figure showing the full majority rule consensus tree from the 5-gene maximum likelihood bootstrap analysis. [file 1471-2148-9-61-S7.pdf]
